# Proteome changes of sheep rumen epithelium during postnatal development

**DOI:** 10.3389/fgene.2022.1031707

**Published:** 2022-10-25

**Authors:** Kaizhi Zheng, Liangyong Guo, Saif Ullah, Yang Cao, Xin Huang, Huili shan, Junfang Jiang, Jianliang Wu, Yongqing Jiang

**Affiliations:** ^1^ Institute of Animal Husbandry and Veterinary, Zhejiang Academy of Agricultural Sciences, Hangzhou, China; ^2^ Huzhou Academy of Agricultural Sciences, Huzhou, China; ^3^ Faculty of Veterinary and Animal Sciences, Lasbela University of Agriculture Water and Marine Sciences, Lasbela, Pakistan

**Keywords:** proteome, sheep, rumen, epithelium, postnatal development

## Abstract

**Background:** The development of the rumen epithelium is a critical physiological challenge for sheep. However, the molecular mechanism underlying postnatal rumen development in sheep remains rarely understood.

**Results:** Here, we used a shotgun approach and bioinformatics analyses to investigate and compare proteomic profiles of sheep rumen epithelium tissue on day 0, 15, 30, 45, and 60 of age. A total of 4,523 proteins were identified, in which we found 852, 342, 164, and 95 differentially expressed proteins (DEPs) between day 0 and day 15, between day 15 and day 30, between day 30 and day 45, between day 45 and day 60, respectively. Furthermore, subcellular localization analysis showed that the DEPs were majorly localized in mitochondrion between day 0 and day 15, after which nucleus proteins were the most DEPs. Finally, Gene Ontology (GO) and Kyoto Encyclopedia of Genes and Genomes (KEGG) analyses showed that DEPs significantly enriched in mitochondrion, ubiquitination, histone modifications, glutathione synthase activity, and wnt and nortch signaling pathways.

**Conclusion:** Our data indicate that the biogenesis of mitochondrion in rumen epithelial cell is essential for the initiation of rumen epithelial development. Glutathione, wnt signaling pathway and nortch signaling pathway participated in rumen epithelial growth. Ubiquitination, post-translational modifications of histone might be key molecular functions in regulating rumen epithelial development.

## 1 Introduction

Sheep are a major source of meat, milk and fiber that have a specialized digestive organ, the rumen (Jiang et al., 2014). Rumen is the most important digestive organ for ruminant animal, which accounts for up to 80% of the energetic needs of mature animals (Bergman, 1990). However, the physiological structure and function of fetal rumen are not well developed during prenatal stage (Arias et al., 1978). For lamb, the transition of milk to solid diets requires the development of a functional rumen. Hence, a fully developed rumen is critical for sheep production.

After birth, the rumen undergoes a series of dramatic ruminal transformations making it to a ruminant, which uses microbial flora to ferment the feed and generates short-chain fatty acid (SCFAs) (Baldwin, 1998). During this transition, the rumen development is consists of three aspects, including morphological development, microbial colonization and functional achievements. For functional achievements, about 85% of SCFAs are directly absorbed by rumen epithelia, facilitating the conversion of low value lignocellulose-rich plant materials to animal protein (Wolin, 1981). While the rumen epithelia also changes to tongue-shaped papillae constituted by stratum basale, stratum spinosum, stratum granulosum and stratum corneum in morphological development, in which stratum spinosum cells played crucial roles in SCFAs metabolism and immune response (Yuan et al., 2022). Followed by the morphological changes and functional achievements of rumen epithelia, great biochemical changes occurs during this stage (Diao et al., 2019), which make rumen basale cell differentiates and migrates to the other types of cells (Steele et al., 2016).

Our previous study has described the dramatic histological changes of lamb rumen papillae from birth to 60 days of age (Zheng et al., 2021). However, we still lack an overview of the changes in protein expression, regulatory factors and protein networks throughout sheep rumen epithelium postnatal development. Recently, proteomics have become a powerful post-genomic tool widely applied to identify and quantify overall proteins present content of a cell, tissue or an organism (Aslam et al., 2017; Keerthikumar, 2017). More and more biologists are using proteomics to reveal single-cell variations, dynamic protein translocations, interaction networks and proteins localizing to multiple compartments (Lundberg and Borner, 2019). Given the central role of rumen epithelium in sheep production, we were promoted to use proteomic as a powerful tool to illuminate the underlying molecular mechanisms that involved in rumen epithelium postnatal development.

## 2 Material and methods

### 2.1 Animals

Male Hu Sheep lamb were randomly selected and fed by ewe. From day 10 of age, pellet was used as supplement for lamb. The lamb were sacrificed either at day 0, day 15, day 30, day 45 or day 60 of age with 3 sheep in each age. Slaughtering was carried out through the assembly line of a local commercial abattoir, the slaughtering method followed the traditional procedures, which include stunning. Rumen epithelia were isolated and frozen in liquid nitrogen. Experimental protocols for animal research were approved by the Institutional Animal Care and Use Committees at the Zhejiang Academy of Agricultural Sciences.

### 2.2 Sample preparation and trypsin digestion for label-free proteome

The sample preparation of protein was performed essentially as the procedure described previously (Liao et al., 2022). Rumen epithelium samples were ground individually in liquid nitrogen and lysed with PASP lysis buffer (100 mM NH4HCO3, 8 M Urea, pH 8.0), followed by 5 min of ultrasonication on ice. The lysate was centrifuged at 12,000 g for 15 min at 4°C. The supernatants were collected and quantified by BCA Protein Assay Kit (Beyotime Institute of Biotechnology, Shanghai, China). 20 μg of the protein sample was loaded to 12% SDS-PAGE gel electrophoresis for quality control and the supernatant was reduced with 10 mM DTT for 1 h at 56°C, and subsequently alkylated with sufficient iodoacetamide (IAM) for 1 h at room temperature in the dark. Then samples were completely mixed with 4 times volume of precooled acetone by vortexing and incubated at −20°C for at least 2 h. Samples were then centrifuged at 12,000 g for 15 min at 4°C and the precipitation was collected.

After washing with 1 ml cold acetone, the pellet was dissolved by dissolution buffer (8 M Urea, 100 mM TEAB, pH 8.5). trypsin and 100 mM TEAB buffer were added, sample was mixed and digested at 37°C for 4 h. Then trypsin and CaCl2 were added digested overnight. Formic acid was mixed with digested sample, adjusted pH under 3, and centrifuged at 12,000 g for 5 min at room temperature. The supernatant was slowly loaded to the C18 desalting column, washed with washing buffer (0.1% formic acid, 3% acetonitrile) 3 times, then added elution buffer (0.1% formic acid, 70% acetonitrile). The eluents of each sample were collected and lyophilized.

### 2.3 LC-MS/MS analysis

LC-MS/MS analysis was then performed according to the procedure described previously (Zhou et al., 2019). Mobile phase A (0.1% formic acid in H2O) and B solution (0.1% formic acid in 80% acetonitrile) were prepared. The lyophilized powder was dissolved in 10 μl of solution A, centrifuged at 14,000 g for 20 min at 4°C, and 1 μg of the supernatant was injected into a home-made C18 Nano-Trap column (4.5 cm × 75 μm, 3 μm). Peptides were separated in a home-made analytical column (15 cm × 150 μm, 1.9 μm), using a linear gradient elution.

LC-MS/MS analyses were performed by using Q ExactiveTM series mass spectrometer (Thermo Fisher, Germany) at Novogene Co., Ltd. (Beijing, China), with ion source of Nanospray Flex™ (ESI), spray voltage of 2.1 kV and ion transport capillary temperature of 320°C. Full scan range from m/z 350 to 1,500 with resolution of 60,000 (at m/z 200), an automatic gain control target value was 3 × 106 and a maximum ion injection time was 20 m s. The top 40 precursors of the highest abundant in the full scan were selected and fragmented by higher energy collisional dissociation and analyzed in MS/MS, where resolution was 15,000 (at m/z 200), the automatic gain control target value was 1 × 105, the maximum ion injection time was 45 m s, a normalized collision energy was set as 27%, an intensity threshold was 2.2 × 104, and the dynamic exclusion parameter was 20 s. The raw data of MS detection was named as “raw”.

### 2.4 Protein identification and quantitation

The all resulting spectra were searched by Proteome Discoverer 2.2 (PD 2.2, Thermo). The corresponding proteins were matched with ovis aries database. The search parameters are set as follows: mass tolerance for precursor ion was 10 ppm and mass tolerance for product ion was 0.02 Da. Carbamidomethyl was specified as fixed modifications, Oxidation of methionine was specified as dynamic modification, and acetylation was specified as N-Terminal modification. A maximum of two missed cleavage sites were allowed. To improve the quality of analysis results, the software PD 2.2 further filtered the retrieval results: Peptide Spectrum Matches (PSMs) with a credibility of more than 99% was identified PSMs. The identified protein contains at least 1 unique peptide. The identified PSMs and protein were retained and performed with FDR no more than 1.0%. Principal component analysis (PCA) and volcano plot, which combined fold-change analysis and t-tests, were performed. Proteins with a minimum fold change of 2 (ratio >2 or <0.5, *p* < 0.05) were considered to be regulated differently between comparisons.

### 2.5 Bioinformatics analysis

GO functional analysis was conducted using the interproscan program against the non-redundant protein database (including Pfam, PRINTS, ProDom, SMART, ProSite, PANTHER), and the databases of Clusters of Orthologous Groups and KEGG (http://www.genome.jp/kegg/) were used to analyze the protein family and pathway. DPEs were used for Volcanic map analysis, cluster heat map analysis and enrichment analysis of GO, subcellular localization and KEGG. The probable protein-protein interactions were predicted using the STRING-db server (http://string.embl.de/).

## 3 Results

### 3.1 Protein identification and comparison analysis

**FIGURE 1:**
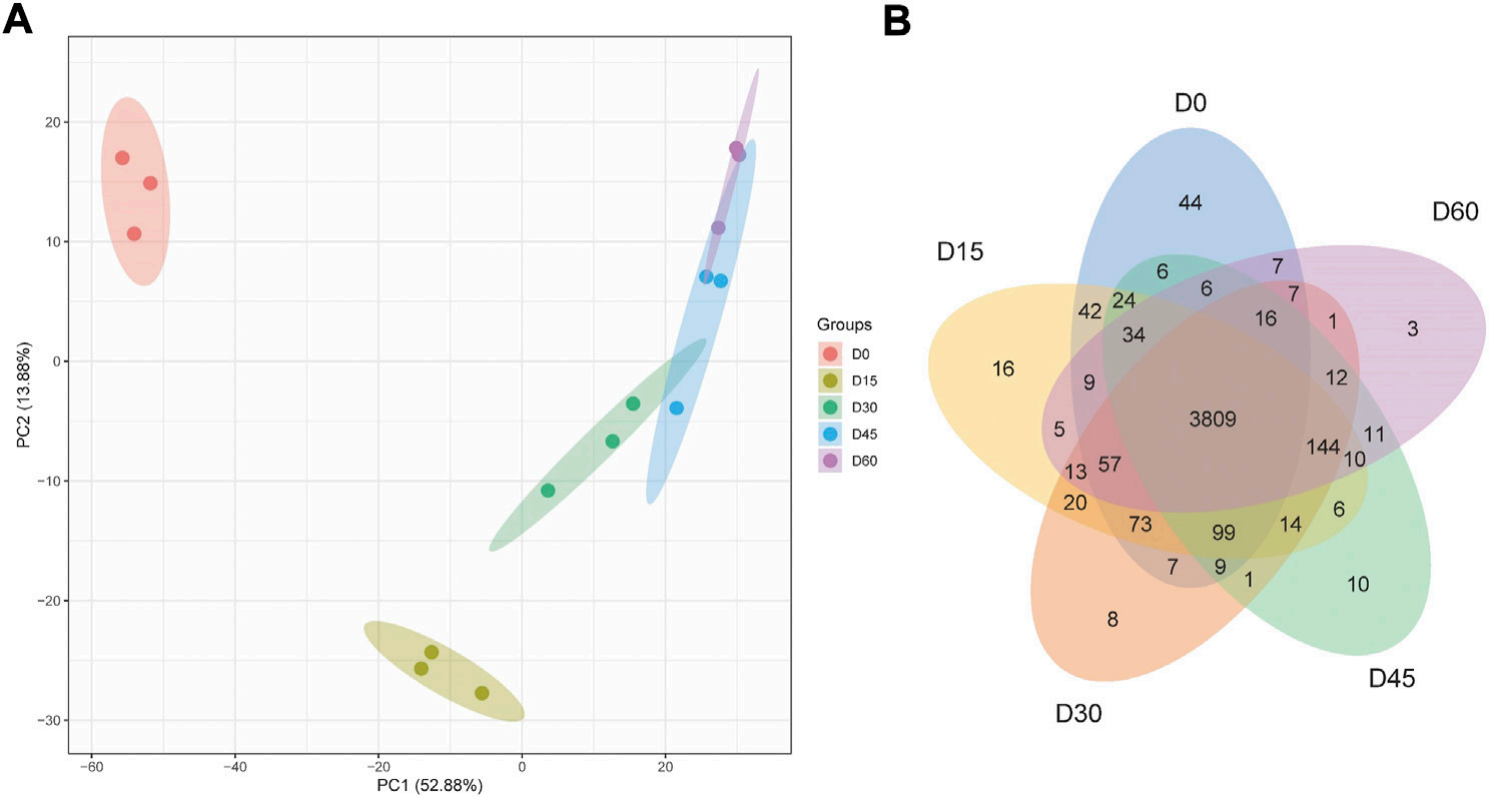
PCA scores plot **(A)** and Venn Diagram **(B)** of proteins identified in rumen epithelium at the age of day 0, day 15, day 30, day 45, and day 60.

To explore the mechanism driving rumen papillae development, we used label-free proteomic strategy to identify different abundant proteins of day 0, day 15, day 30, day 45, and day 60. A total of 4,523 proteins were identified, including 44 proteins uniquely expressed in day 0, 16 proteins uniquely expressed in day 15, 8 proteins uniquely expressed in day 30, 10 proteins uniquely expressed in day 45 and 3 proteins uniquely expressed in day 60 (Figure 1B). To visually differentiate the sample clusters among the observations, PCA analysis was performed to compare the mutual proteins among rumen epithelium tissues of different ages. We found that 66.76% of the variability was explained by the first two principal components, which accounted for 13.88%, and 52.88% of the total variance. The rumen epithelia of day 0, day 15, day 30, and day 60 could be separated completely by identified proteins and distributed in different locations. However, the rumen epithelia of day 45 could not be completely separated from that of day 30 and day 60 (Figure 1A).

**FIGURE 2:**
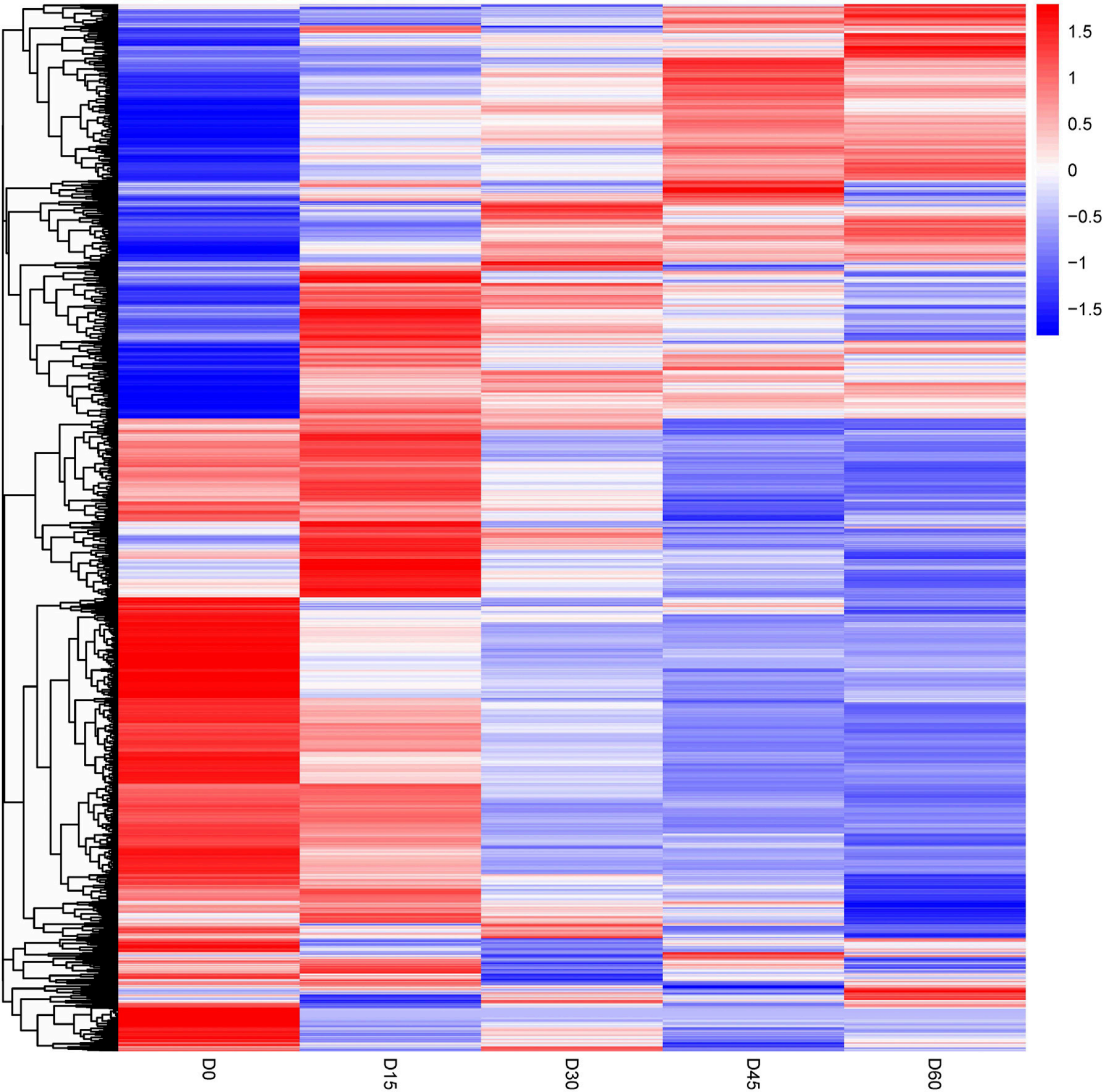
Heat map of differentially abundant proteins in rumen epithelium at the age of day 0, day 15, day 30, day 45 and day 60.

The differentially abundant proteins were highlighted by simultaneously considering fold change >2 or <0.5 and *p* < 0.05. A total of 852 proteins were significantly different in comparison of day 0 and day 15 rumen epithelia (Supplementary Table S1). While the number of DEPs was 342 between rumen epithelia of day 15 and day 30 (Supplementary Table S2). Afterwards, this number became 164 between day 30 and day 45 (Supplementary Table S3). And finally, the number DEPs was 95 between rumen epithelia of day 45 and day 60 (Supplementary Table S4). Unsupervised hierarchical clustering of different biological data sets was then conducted as a more rigorous test for the screened proteins to evaluate the rationality, accuracy and dynamic changes of these DEPs (Figure 2).

### 3.2 Subcellular localization of the DEPs

**FIGURE 3:**
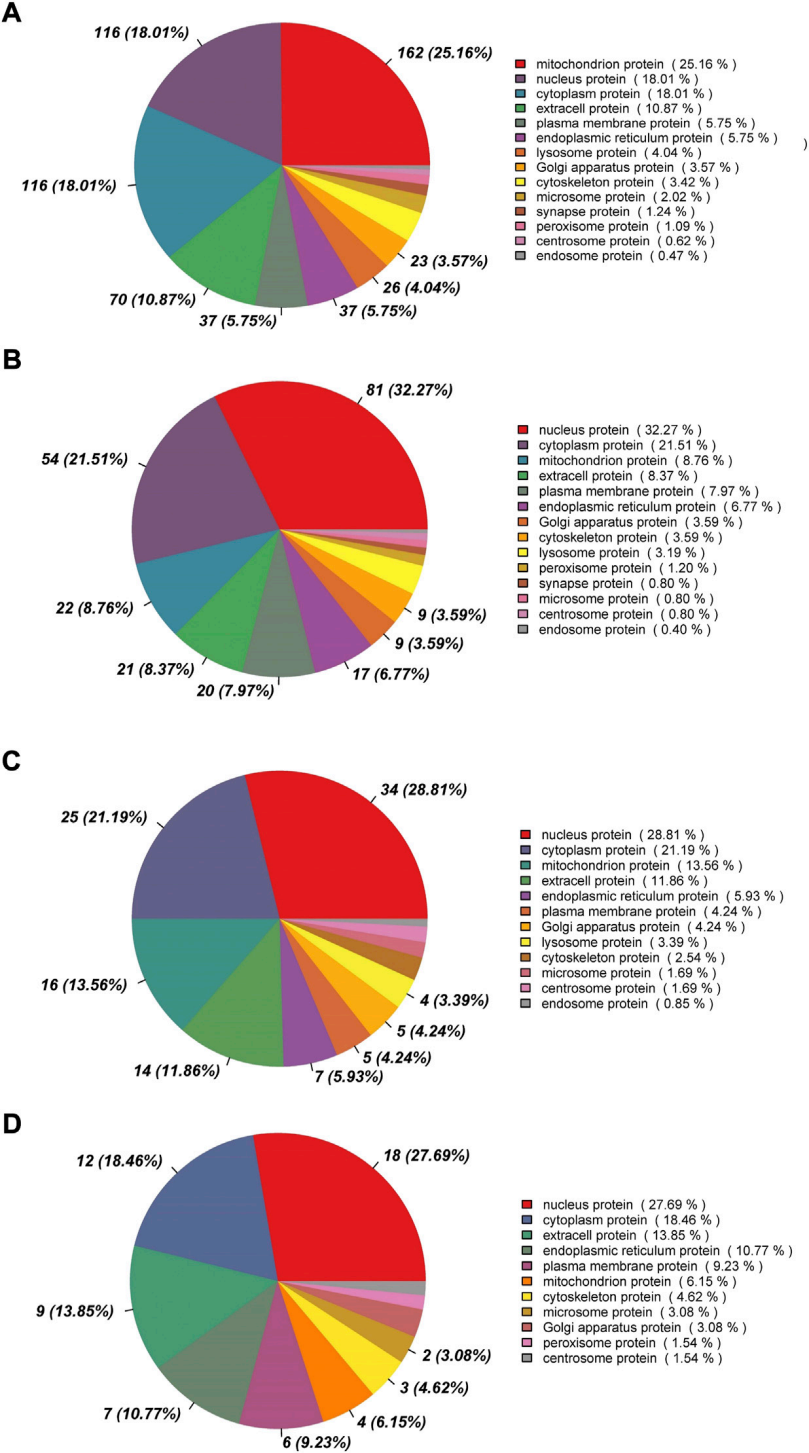
Subcellular localization analysis of differentially abundant proteins in rumen epithelium between **(A)** day 0 and day 15, **(B)** day 15 and day 30, **(C)** day 30 and day 45 and **(D)** day 45 and day 60 of age.

Between rumen epithelia of day 0 and day 15%, 25.16% of the DEPs were mitochondrion proteins, followed by nucleus proteins (18.01%) and cytoplasm proteins (18.01%) (Figure 3A). Between day 15 and day 30, the majority of DEPs were localized in nucleus (32.27%), while 21.51% were cytoplasm proteins and only 8.76% were mitochondrion proteins (Figure 3B). The DEPs between day 30 and day 45 of age contained 28.81% nucleus proteins, 21.19% cytoplasm proteins and 13.56% mitochondrion proteins (Figure 3C). While Between day 0 and day 15, it contained 27.69% nucleus proteins, 18.46% cytoplasm proteins and 13.85% extracell proteins (Figure 3D).

### 3.3 Bioinformatics analysis of differentially abundant proteins

**FIGURE 4:**
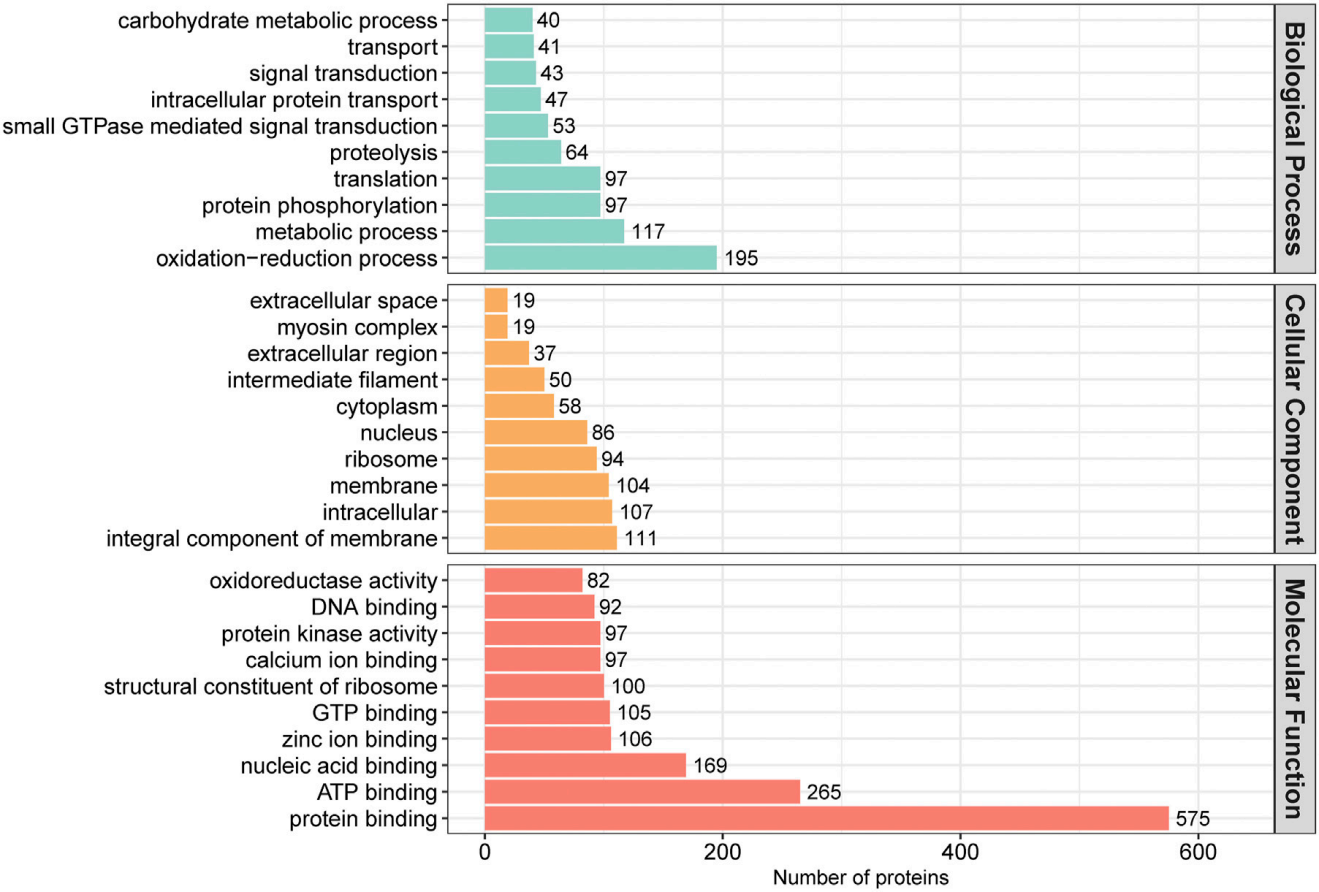
GO annotation classification of differentially abundant proteins in rumen epithelium at the age of day 0, day 15, day 30, day 45 and day 60.

**FIGURE 5:**
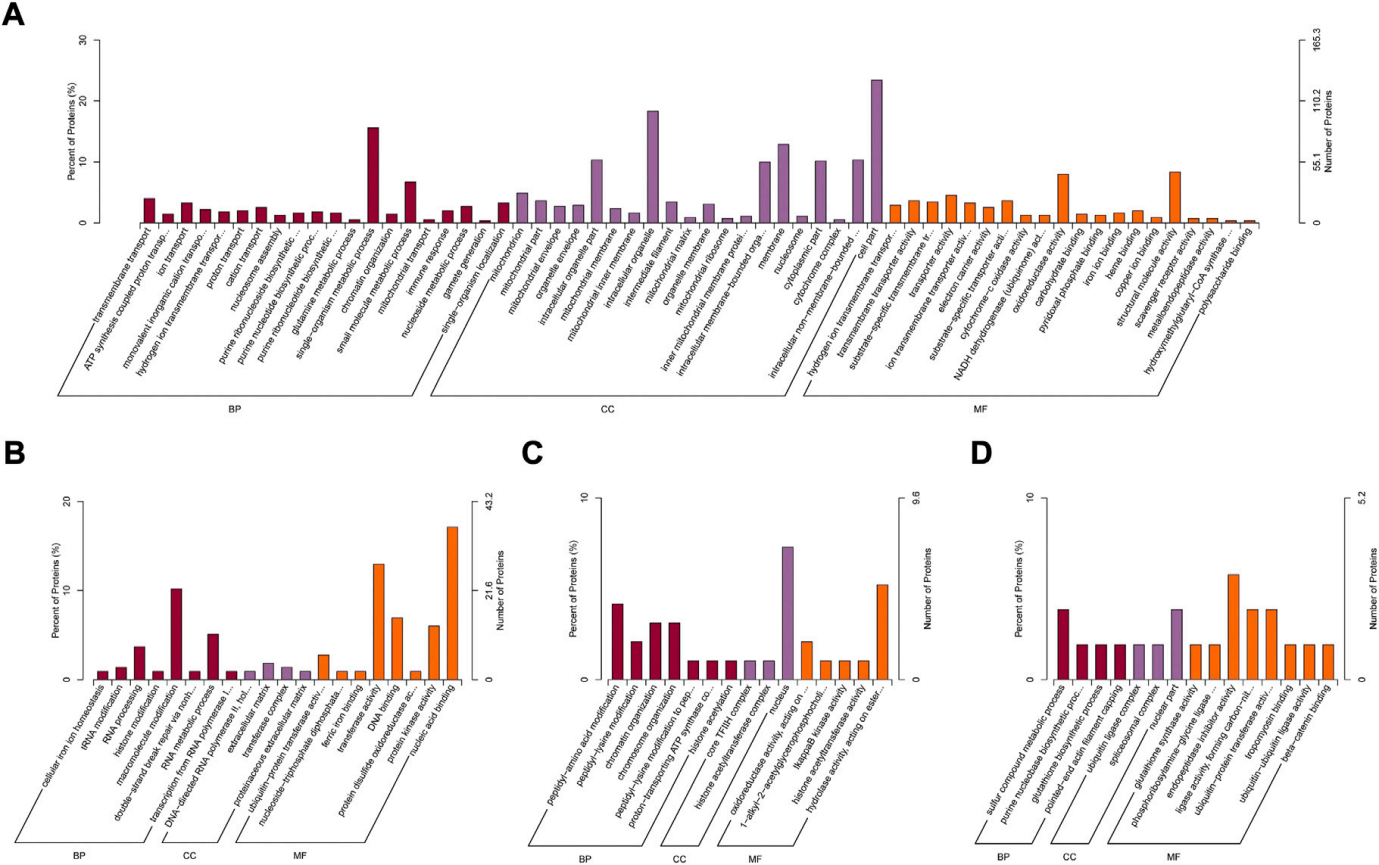
GO enrichment analysis of differentially abundant proteins in rumen epithelium between **(A)** day 0 and day 15, **(B)** day 15 and day 30, **(C)** day 30 and day 45 and **(D)** day 45 and day 60 of age. All pathways are listed according to *p*-value.

In order to assess the major biological processes that participated in rumen epithelial development, we perform a GO annotation to analyze the functional characteristics of proteins identified in sheep rumen epithelia of day 0, day 15, day 30, day 45, and day 60 (Figure 4). Bioinformatics analyses were then performed to construct a specific molecular network to explore the biological functions and pathways related to the DEPs from birth to 60 days of age. GO enrichment analysis showed that DEPs in rumen epithelia were significantly enriched in 84 GO terms between day 0 and day 15 (*p* < 0.05). In the biological processes analysis, the top five biological progresses were transmembrane transport (GO:0055085), ATP synthesis coupled proton transport (GO:0015986), ion transport (GO:0006811), monovalent inorganic cation transport (GO:0015672) and hydrogen ion transmembrane transport (GO:1902600). In molecular functions, the top five significant GO terms were hydrogen ion transmembrane transporter activity (GO:0015078), transmembrane transporter activity (GO:0022857), substrate-specific transmembrane transporter activity (GO:0022891), transporter activity (GO:0005215) and ion transmembrane transporter activity (GO:0015075). As for cell components, mitochondrion (GO:0005739), mitochondrial part (GO:0044429), mitochondrial envelope (GO:0005740), organelle envelope (GO:0031967) and intracellular organelle part (GO:0044446) were the top five GO terms that enriched significantly (*p* < 0.05) (Figure 5A, Supplementary Table S5).

While the number of GO enriched terms decreased to 20 between day 15 and day 30 of age. The top five molecular function GO terms were ubiquitin-protein transferase activity (GO:0004842), nucleoside-triphosphate diphosphatase activity (GO:0047429), ferric iron binding (GO:0008199), transferase activity (GO:0016740) and DNA binding (GO:0003677). As for biology processes, cellular iron ion homeostasis (GO:0006879), tRNA modification (GO:0006400), RNA processing (GO:0006396), histone modification (GO:0016570) and macromolecule modification (GO:0043412) were the top five significantly enriched GO terms. And for cell components, the significantly enriched GO terms included DNA-directed RNA polymerase II, holoenzyme (GO:0016591), extracellular matrix (GO:0031012), transferase complex (GO:1990234) and proteinaceous extracellular matrix (GO:0005578) (*p* < 0.05) (Figure 5B, Supplementary Table S6).

Between rumen epithelium tissues of day 30 and day 45, the significantly enriched GO terms of biology progresses were peptidyl-amino acid modification (GO:0018193), peptidyl-lysine modification (GO:0018205), chromatin organization (GO:0006325), chromosome organization (GO:0051276), peptidyl-lysine modification to peptidyl-hypusine (GO:0008612), proton-transporting ATP synthase complex assembly (GO:0043461) and histone acetylation (GO:0016573). As for molecular functions, the GO terms included oxidoreductase activity (GO:0016705), 1-alkyl-2-acetylglycerophosphocholine esterase activity (GO:0003847), IkappaB kinase activity (GO:0008384), histone acetyltransferase activity (GO:0004402) and hydrolase activity (GO:0016788). In cell components, DEPs were enriched in core TFIIH complex (GO:0000439), histone acetyltransferase complex (GO:0000123) and nucleus (GO:0005634) (*p* < 0.05) (Figure 5C, Supplementary Table S7).

Between rumen epithelium tissues of day 45 and day 60, the significantly enriched GO terms of biology progresses were sulfur compound metabolic process (GO:0006790), purine nucleobase biosynthetic process (GO:0009113), glutathione biosynthetic process (GO:0006750) and pointed-end actin filament capping (GO:0051694). As for molecular functions, the GO terms included glutathione synthase activity (GO:0004363), phosphoribosylamine-glycine ligase activity (GO:0004637), endopeptidase inhibitor activity (GO:0004866), ligase activity, forming carbon-nitrogen bonds (GO:0016879) and ubiquitin-protein transferase activity (GO:0004842), tropomyosin binding (GO:0005523), ubiquitin-ubiquitin ligase activity (GO:0034450) and beta-catenin binding (GO:0008013). In cell components, DEPs were enriched in ubiquitin ligase complex (GO:0000151), spliceosomal complex (GO:0005681) and nuclear part (GO:0044428) (*p* < 0.05) (Figure 5D, Supplementary Table S8).

### 3.4 kyoto encyclopedia of genes and genomes pathway enrichment analysis

**FIGURE 6:**
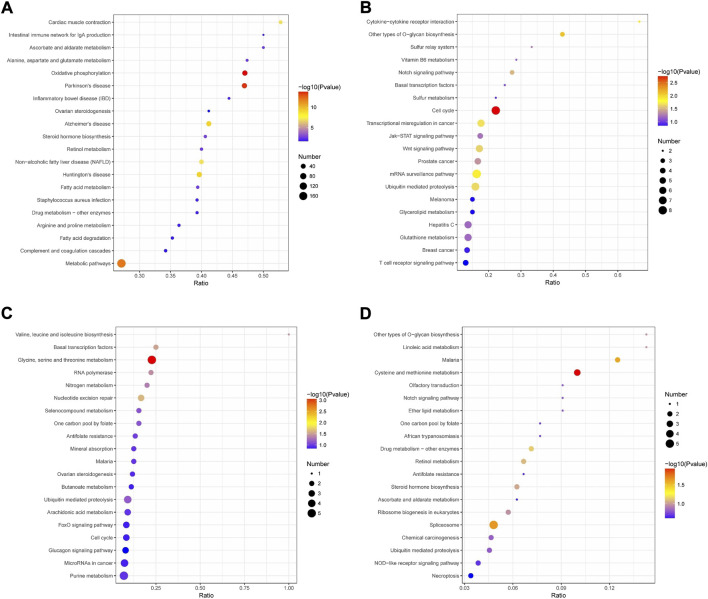
KEGG pathway enrichment analysis of differentially abundant proteins in rumen epithelium between **(A)** day 0 and day 15, **(B)** day 15 and day 30, **(C)** day 30 and day 45 and **(D)** day 45 and day 60 of age. All pathways are listed according to *p*-value.

Then, KEGG pathway enrichment was performed to extract the biological pathways related to the differentially abundant proteins in rumen epithelia from birth to day 60 of age. Between day 0 and day 15, the DEPs were significantly enriched in 33 pathways. The top five significantly enriched KEGG terms were oxidative phosphorylation, Parkinson’s disease, metabolic pathways, Huntington’s disease and Alzheimer’s disease (*p* < 0.05)(Figure 6A, Supplementary Table S9). From day 15 to day 30 of age, the number of significantly enriched KEGG pathways decreased to 9, including cell cycle, other types of O-glycan biosynthesis, cytokine-cytokine receptor interaction, mRNA surveillance pathway, transcriptional misregulation in cancer, ubiquitin mediated proteolysis, Wnt signaling pathway, notch signaling pathway and prostate cancer (*p* < 0.05)(Figure 6B, Supplementary Table S10). From day 30 to day 45 of age, the significantly enriched KEGG pathways were glycine, serine and threonine metabolism, nucleotide excision repair, basal transcription factors, valine, leucine and isoleucine biosynthesis, RNA polymerase and nitrogen metabolism (*p* < 0.05) (Figure 6C, Supplementary Table S11). While, from day 45 to day 60 of age, the significantly enriched KEGG pathways were only cysteine and methionine metabolism (map00270), spliceosome (map03040) and malaria (map05144) (*p* < 0.05)(Figure 6D, Supplementary Table S12).

## 4 Discussion

The development of the rumen is a critical physiological challenge in newborn ruminants. A better developed rumen epithelium is important for better feed efficiency, which increases profit for producers. In this study, we obtained a comprehensive landscape of proteome profiles across 15 rumen tissue samples during five stages of lamb sucking period, which provided a system overview of proteomic changes of sheep rumen epithelium from birth to weaning.

The high consistence of PCA and heat map analysis results showed that the DEPs upon different ages were distinguishable among rumen epithelium samples of day 0, day 15, day 30, and day 60. While the results of day 45 could not be distinguished from that of day 30 and day 60. Our previous study has found significant histological changes occur on sheep rumen epithelium after birth, which forms tongue shaped papillae at the age of day 15 (Zheng et al., 2021). These data also indicate great biochemical changes occur from birth to day 15 of age in rumen epithelial cells, which might result from dramatic protein expression changes. Consistent with our previous study, 621 proteins were up-regulated and 231 proteins were down-regulated on day 15 of age. After GO and KEGG enrichment analysis, we found that oxidative phosphorylation (OXPHOS) and proton transport were important functions that rumen epithelium gained or enhanced during this period.

The cells of the stratum basale contain mitochondria that contribute to the metabolic properties of the ruminal stratified squamous epithelia (Graham and Simmons, 2005). Currently, researchers have uncovered an important role for mitochondria in the differentiation of the neuronal, hematopoetic, mesenchymal and cardiac systems (Piccoli et al., 2005; Chung et al., 2007; Chen et al., 2008; Khacho et al., 2016). Mitochondrion is a complex organelle that plays essential roles in energy transduction, ATP production and cellular signaling events. In stem cells, differentiation is usually accompanied by a metabolic switch and activation of mitochondrial respiration resulting in the shift from glycolysis to oxidative phosphorylation (Shyh-Chang et al., 2013). Normal differentiated cells primarily rely on mitochondrial OXPHOS to generate the energy for cellular proliferation (Vander Heiden et al., 2009). In rumen epithelium, mitochondria is considered to play a crucial role in the metabolism of short-chain fatty acid by producing ketone bodies, an important circulating source of energy (Leighton et al., 1983). In GO enrichment analysis between day0 and day15 group, we found that mitochondrion and mitochondrial part were top two significantly enriched GO terms, in which more than twenty proteins up-regulated in day 15 of age. While, in KEGG analysis, we also found the up-regulated proteins in rumen epithelia of day 15 significantly enriched in OXPHOS, metabolic pathways, fatty acid metabolism and biosynthesis and citrate cycle when comparing with that of day 0. The subcellular localization analysis also showed that the majority of DEPs were mitochondrion proteins during this stage. These results indicated that the biogenesis of mitochondrion plays a key role in the early development of rumen epithelia. Our results also indicated that the establishment of mitochondrial function in rumen epithelial cell was essential for the initiation of rumen epithelial development. And sheep rumen epithelium might start to gain its function on fatty acid metabolism after the age of day 15.

Proton transport is involved in cellular functions like generation of H+ gradients (Kuijpers and De Pont, 1987). In mammals, generation H+ gradients is of critical importance for mitochondrial energy transduction across mitochondrial inner membrane (Verkhovskaya and Bloch, 2013). Mitochondrion moves protons across its inner membrane during OXPHOS, which converts the energy into high energy phosphates contained within newly generated ATP molecules (Poburko and Demaurex, 2012). Hence, establishment of the proton gradient across the mitochondrial inner membrane is the basis of OXPHOS (Ikon and Ryan, 2017). However, a small proportion of protons directly flow into the mitochondrial matrix across inner mitochondrial membrane without generating ATP, which is called proton leaking (Nanayakkara et al., 2019). Mitochondria are a major source of cellular reactive oxygen species (ROS), which is known as a signaling molecule in various of physiological or pathological conditions (Zhao et al., 2019). The amount of generated ROS in mitochondria determines whether ROS play beneficial or harmful roles. Mitochondrial membrane potential is highly correlated with ROS production, while proton leaking can inhibit the production of ROS by reducing mitochondrial membrane potential (Negre-Salvayre et al., 1997; Suski et al., 2018). Previous studies have reported that proton leaking plays a protective role in early neuronal development (Hoang et al., 2012). Our results showed that most of the significantly enriched GO terms has either direct or indirect relationship with mitochondrial ion transmembrane transporter activity, especially proton transport, between rumen epithelia of day 0 and day 15. In KEGG analysis between rumen epithelia of day 0 and day 15, we also found DEPs significantly enriched in neurodegenerative disease that related to mitochondria and ROS, like Parkinson’s disease, Huntington’s disease and Alzheimer’s disease (Kim-Han and Dugan, 2005; Jodeiri Farshbaf and Ghaedi, 2017; Tonnies and Trushina, 2017; Shen et al., 2020). The expression of these disease related proteins decreased when proton transport proteins up-regulated on day 15 of age, indicating a protective role of proton transport system in mitochondria of rumen epithelial cell during early rumen development.

In GO enrichment analysis, we found the most significant GO term between rumen epithelia of day 15 and day 30 significantly was ubiquitin-protein transferase activity. Ubiquitination is one of the post-translational modifications that target lysine residue and regulate many cellular processes, including cell division, cell differentiation and immune responses (Popovic et al., 2014). The expression of E3 ubiquitin-protein ligases were down-regulated on day 30. In KEGG enrichment analysis, we also found significant enriched pathways of cell cycle, wnt signaling pathway and notch signaling contained E3 ubiquitin-protein ligases. Wnt signaling is a critical component during embryonic development (Tran and Zheng, 2017), while notch signaling regulates many aspects of metazoan development and tissue renewal (Kopan and Ilagan, 2009). These evidence indicated that rumen epithelial development started since this stage, both wnt signaling and notch signaling were important pathways that participated in this process. And ubiquitination might be a key molecular function in regulating rumen epithelial development.

Post-translational modifications of histone are known to be essential for the regulation of gene transcription, which affect a wide range of biological processes. Histone acetylation is well known as an important regulator of gene expression, which permits switching between permissive and repressive chromatin upon sensing developmental and environmental cues (Shen et al., 2015). Histone acetylation level is controlled by the activity of histone acetyltransferase and deacetylase. The biology function of histone acetyltransferase include cell proliferation, DNA damage repair and glucose metabolism (Poziello et al., 2021). Our previous study has found that the rumen papillae already formed layers of stratum corneum on day 30 of age, while there is no significant histological change except the length of papillae since then (Zheng et al., 2021). In this study, we found DEPs significantly enriched in histone acetylation, and the expression of histone acetyltransferase significantly decreased on day 45 of age. These results indicated that histone acetylation was an important biology process in modulating rumen epithelial development.

The Hu Sheep is naturally weaned around day 60 of age, implying that the function of rumen epithelium has been fully established. In GO enrichment analysis between rumen epithelia of day 45 and day 60, the DEPs were significantly enriched in glutathione synthase activity and glutathione synthetase was up-regulated on day 60 of age. Glutathione is a ubiquitous low molecular weight thiol in eukaryotes, which is required for a diverse range of processes including growth, stress tolerance and cell suicide programs (Diaz-Vivancos et al., 2015). On one hand, it is recruited into the nucleus in early phases of cell proliferation for the synthesis of DNA (Markovic et al., 2007). On the other hand, glutathione is known to remove ROS, which is required for proliferation in response to insulin, fibroblast growth factor, platelet derived growth factor, epidermal growth factor and other growth factors (Burch and Heintz, 2005). These evidence indicated a decrease of rumen epithelial cell proliferation, which is consistent with our previous finding that the ratio of Ki67 positive cell significantly decrease on day 60 of age (Zheng et al., 2021).

To conclude our data indicate that the biogenesis of mitochondrion in rumen epithelial cell is essential for the initiation of rumen epithelial development. Glutathione, Wnt signaling pathway and Nortch signaling pathway participated in rumen epithelial growth. And ubiquitination and post-translational modifications of histone might be key molecular function in regulating rumen epithelial development. The proposed datasets provide a useful basis for future studies to better comprehend rumen epithelial development.

## Data Availability

The data presented in the study are deposited in the ProteomeXchange Consortium repository, accession number PXD034787.
